# Association of triglyceride-glucose-related indices with adverse clinical outcomes in individuals with normal body mass index

**DOI:** 10.3389/fcvm.2025.1570239

**Published:** 2025-04-24

**Authors:** Jiejie Xie, Xiong Pei, Shixuan Zhu, Wei Jiang, Hong Tang, Dongbo Wu, Yan Xie

**Affiliations:** ^1^ Department of Gastroenterology, West China Hospital, Sichuan University, Chengdu, Sichuan, China; ^2^ Center of Infectious Diseases, West China Hospital, Sichuan University, Chengdu, Sichuan, China

**Keywords:** TyG index, liver fibrosis, stroke, cardiovascular disease, mortality

## Abstract

**Background and aims:**

The triglyceride-glucose (TyG) index serves as a reliable indicator of insulin resistance and metabolic risk factors. Most research has focused on obese individuals, with limited exploration in those with a normal body mass index (BMI).

**Method:**

This study analyzed 4,440 adults with normal BMI from NHANES 2003–2018. Logistic regression, linear regression, subgroup analysis, and survival analysis examined the relationship between TyG-related indices (TyG, TyG-BMI, TyG-WC, TyG-WHtR) and outcomes like liver fibrosis, stroke, cardiovascular disease (CVD), and mortality.

**Results:**

In 4,440 individuals, 279 developed CVD, 134 had stroke, 1,382 developed liver fibrosis, and 548 died, with a median observation period of 100 months (IQR, 59–145 months). The TyG index was divided into four quartiles (Q1, Q2, Q3, Q4) and significant trends in various clinical indicators across the quartiles were observed (demographic characteristics, metabolic and biochemical indicators). Further analysis revealed linear correlations between TyG, TyG-WC, TyG-BMI, TyG-WHtR and liver function metrics (ALT, AST, GGT, FIB-4, APRI), kidney function metrics (creatinine, eGFR, uric acid), and blood lipids (triglycerides, cholesterol) (*P* < 0.01). Univariate logistic regression showed that compared to Q1, Q4 showed a significantly higher risk of liver fibrosis, CVD, stroke, and death for all TyG-related parameters (*P* < 0.001). After adjusting for cofounders, TyG Q4 still had a significantly higher risk of liver fibrosis (*P* < 0.05) and mortality (*P* < 0.001); TyG-BMI Q4 showed a higher risk of mortality (*P* < 0.001); TyG-WC Q4 showed a significantly higher risk of liver fibrosis (*P* < 0.001), stroke (*P* < 0.01), CVD (*P* < 0.001), and mortality (*P* < 0.001); TyG-WHtR Q4 showed a significantly higher risk of liver fibrosis (*P* < 0.001), stroke (*P* < 0.01), CVD (*P* < 0.001), and mortality (*P* < 0.001). Subgroup analysis yielded similar conclusions. Additionally. Survival analysis revealed significant differences in survival across the different quartiles of TyG, TyG-WC, TyG-BMI, and TyG-WHtR (*P* < 0.001).

**Conclusion:**

The study identified a link between TyG-related markers and negative outcomes in individuals with a normal BMI, indicating that insulin resistance exists even in non-obese populations.

## Introduction

Insulin resistance (IR) is widely recognized as a key factor in the development of various metabolic disorders, including type 2 diabetes (1, 2), and fatty liver disease (3, 4), and cardiovascular diseases (5, 6). Additionally, IR is also known to be associated with a range of adverse clinical outcomes, such as increased risk of stroke (7, 8), myocardial infarction (9, 10), and all-cause mortality (11). Early identification of IR is important for the prevention and management of these conditions.

IR is often asymptomatic in its early stages. Conventional approaches for diagnosing insulin resistance, such as the hyperinsulinemic-euglycemic clamp and the homeostasis model assessment of insulin resistance (HOMA-IR) (12), are intricate, costly, and not commonly applied in everyday clinical settings. In recent times, the triglyceride-glucose (TyG) index has become recognized as an important alternative marker, providing a straightforward and reliable measure of insulin resistance (13, 14). It has been demonstrated that the TyG index exhibits a strong correlation with insulin resistance, making it a potentially valuable for identifying individuals at risk for metabolic disorders. Zeng et al. revealed that TyG index correlates positively with the gestational diabetes mellitus (15). Guo et al. found that people with a higher TyG index are more likely to experience reduced cardiovascular fitness (16). Variations of the TyG index, such as TyG-BMI (body mass index), TyG-WC (waist circumference), and TyG-WHtR (waist-to-height ratio), have been introduced to enhance the evaluation of insulin resistance by integrating various body metrics. The TyG index and its related parameters have been predominantly studied in populations with overweight or obesity, where the prevalence of insulin resistance is high. However, emerging evidence suggests that insulin resistance is also present in individuals with a normal BMI, especially in those with visceral fat accumulation or other risk factors that may not be captured by BMI alone (17–19).

In light of this, the objective of the current study is to investigate the relationship between TyG-related indices (TyG, TyG-BMI, TyG-WC, and TyG-WHtR) and clinical outcomes, such as liver fibrosis, stroke, cardiovascular disease (CVD), and all-cause mortality, in individuals with a normal BMI (18.5 ≤ BMI < 25 kg/m^2^). This research could contribute to a better understanding of insulin resistance in normal-weight populations and inform strategies for early intervention and prevention of related health complications.

## Materials and methods

### Data source and study population

This cross-sectional study included 4,440 adult participants, utilizing data from the National Health and Nutrition Examination Survey (NHANES) covering the period from 2003 to 2018. Exclusion criteria comprised the following: (1) BMI ≥ 25 kg/m^2^; (2) age <20 years; (3) Individuals missing data on the triglyceride-glucose index and its relationship with obesity-related markers; (4) Participants without data on the outcome or covariates were excluded. The NHANES study protocol received approval from the NCHS Research Ethics Review Board, with all participants signing written informed consent forms.

### Formulas of TyG indices

The TyG index is derived from the combination of fasting triglyceride levels and blood glucose. TyG, TyG-BMI, TyG-WC, and TyG-WHtR were calculated according to the following formulas: (1) TyG = ln [triglycerides (mg/dl) × glucose (mg/dl)/2]; (2) TyG-BMI = TyG × BMI [BMI = body mass (kg)/height^2^(m^2^)]; (3) TyG-WC = TyG × waist circumference(cm); (4). TyG-WHtR = TyG × WHtR [WHtR = waist circumference(cm)/height(m)].

### Disease definition

Liver fibrosis was evaluated using the fibrosis-4 (FIB-4) score, calculated as ([age × AST] [platelet count ×]). A FIB-4 value greater than 1.3 was considered indicative of liver fibrosis in individuals with increased liver enzyme levels which were defined as aspartate aminotransferase (AST) or alanine aminotransferase (ALT) exceeding 25 U/L in women and 35 U/L in men (20, 21). Chronic kidney disease (CKD) was defined as estimated glomerular filtration rate (eGFR) ≤60. The diagnosis of CVD, stroke, diabetes, and hypertension was determined based on self-reported physician diagnoses, collected through a standardized medical condition questionnaire during individual interviews. Participants were asked, “Has a doctor or healthcare professional ever diagnosed you with congestive heart failure, coronary artery disease, angina, heart attack, stroke, diabetes, or high blood pressure?”. Individuals were classified as having CVD, stroke, diabetes, or hypertension if they responded “yes” to any of the aforementioned questions.

### Assessment of covariates

Race/ethnicity were categorized into five groups of Mexican American, None-Hispanic Black, None-Hispanic White, Other Hispanic, Other Race. Educational level was categorized into “above high school” and “High school and below” based on the educational survey questionnaire. Smoking was categorized as non-smoking (individuals who had smoked fewer than 100 cigarettes in their lifetime) and smoking (individuals who had smoked 100 or more cigarettes in their lifetime). Drinking was defined as non-drinking (fewer than 12 alcoholic beverages consumed per year) and drinking (12 or more alcoholic beverages consumed annually). Marital status was divided into divorced, living with partner, married, never married, separated, and widowed. Ratio of family income to poverty was divided into the following three intervals: less than 1, 1–4.9, and greater than or equal to 5. Additional information regarding the measurement of covariates is available on the NHANES website (https://www.cdc.gov/nchs/nhanes/?CDC_AAref_Val=https://www.cdc.gov/nchs/nhanes/index.htm).

### Statistical analysis

The statistical analyses incorporated sample weights, clustering, and stratification to address the complex multistage stratified sampling methodology employed in NHANES. The baseline characteristics of participants were analyzed based on the quartiles of the TyG index. Participants were divided into four groups (Q1, Q2, Q3, Q4) according to the quartile distribution of the TyG index. Continuous variables were presented as mean ± standard deviation (SD), normality was evaluated using the Shapiro–Wilk test. Frequencies and percentages were used to describe categorical variables. Differences between groups were assessed with the Kruskal–Wallis test for continuous variables and the chi-square test for categorical variables. Linear regression analysis was used to examine the relationship between TyG-related indices and liver function metrics [ALT, AST, GGT, FIB-4, aspartate transaminase-to-platelet ratio index (APRI)], kidney function metrics (creatinine, eGFR, uric acid), blood lipids (triglycerides, cholesterol). Linear correlation was assessed using Spearman test. The weighted Cox proportional hazards model was applied to calculate odds ratios (ORs) and 95% confidence intervals (CIs) for the relationship between TyG-related indices and outcomes including liver fibrosis, CVD, stroke, and survival. Three models were constructed: Model 1 served as the unadjusted crude model; Model 2 accounted for adjustments involving age, gender, and race; and Model 3 included further adjustments for age, gender, race, BMI, hypertension, diabetes, educational attainment, marital status, family income-to-poverty ratio, smoking, and alcohol consumption. The lowest quartile of TyG-related indices was used as the reference group. At the same time, we conduct subgroup analyses based on gender, educational level, hypertension, diabetes, CKD, and smoking status. Statistical analyses were conducted utilizing SPSS software (version 23.0, IBM Corp., Armonk, NY, USA) and R software (version 4.3.0, The R Foundation, available at http://www.r-project.org).

## Results

### Baseline characteristics of participants according to the quartile of TyG index

The characteristics of the participants were shown in Table 1. People with increased TyG levels were more prone to demonstrate elevated blood glucose, triglycerides, ALT, GGT, and LDH, along with lower albumin, higher globulin, increased cholesterol, elevated uric acid, and greater waist circumference. Moreover, individuals with higher TyG were more likely to develop hypertension, diabetes, CVD, stroke, emphysema, chronic bronchitis, liver diseases, tumors, and CKD. They were also more likely to be smokers and alcohol drinkers. Additionally, TyG was also correlated with education level, marital status, ethnicity and income.

**Table 1:** Baseline characteristics of subjects categorized by TyG index.

Variable		Q1 (*N* = 1,101)	Q2 (*N* = 1,100)	Q3 (*N* = 1,099)	Q4 (*N* = 1,100)	*P*
Gender, *n* (%)	Female	690 (62.7)	597 (54.3)	555 (50.5)	475 (43.2)	<0.001
	Male	411 (37.3)	503 (45.7)	544 (49.5)	625 (56.8)	
Age (mean, SD)		38.98 (16.08)	45.77 (18.83)	49.70 (19.36)	54.00 (18.30)	<0.001
Race, *n* (%)	Mexican American	112 (10.2)	104 (9.5)	131 (11.9)	137 (12.5)	<0.001
	Non-Hispanic Black	279 (25.3)	179 (16.3)	153 (13.9)	112 (10.2)	
	Non-Hispanic White	492 (44.7)	562 (51.1)	560 (51.0)	541 (49.2)	
	Other Hispanic	65 (5.9)	75 (6.8)	78 (7.1)	83 (7.5)	
	Other Race	153 (13.9)	180 (16.4)	177 (16.1)	227 (20.6)	
Height (mean, SD)		167.63 (9.23)	167.73 (9.55)	167.84 (9.93)	167.35 (10.41)	0.679
Weight (mean, SD)		62.17 (8.75)	62.90 (8.90)	63.83 (9.10)	64.62 (9.46)	<0.001
BMI (mean, SD)		22.05 (1.74)	22.27 (1.69)	22.57 (1.70)	22.96 (1.54)	<0.001
Hypertension, *n* (%)	No	969 (88.0)	883 (80.3)	844 (76.8)	715 (65.0)	<0.001
	Yes	132 (12.0)	217 (19.7)	255 (23.2)	385 (35.0)	
Diabetes, *n* (%)	No	1,082 (98.3)	1,054 (95.8)	1,050 (95.5)	910 (82.7)	<0.001
	Yes	19 (1.7)	46 (4.2)	49 (4.5)	190 (17.3)	
Asthma, *n* (%)	No	961 (87.3)	967 (87.9)	988 (89.9)	987 (89.7)	0.131
	Yes	140 (12.7)	133 (12.1)	111 (10.1)	113 (10.3)	
CVD, *n* (%)	No	1,069 (97.1)	1,043 (94.8)	1,030 (93.7)	979 (89.0)	<0.001
	Yes	32 (2.9)	57 (5.2)	69 (6.3)	121 (11.0)	
Stroke, *n* (%)	No	1,081 (98.2)	1,075 (97.7)	1,063 (96.7)	1,047 (95.2)	<0.001
	Yes	20 (1.8)	25 (2.3)	36 (3.3)	53 (4.8)	
Emphysema, *n* (%)	No	1,090 (99.0)	1,081 (98.3)	1,071 (97.5)	1,064 (96.7)	0.002
	Yes	11 (1.0)	19 (1.7)	28 (2.5)	36 (3.3)	
Chronic bronchitis, *n* (%)	No	1,068 (97.0)	1,067 (97.0)	1,053 (95.8)	1,045 (95.0)	0.034
	Yes	33 (3.0)	33 (3.0)	46 (4.2)	55 (5.0)	
Cancer, *n* (%)	No	1,037 (94.2)	1,014 (92.2)	978 (89.0)	977 (88.8)	<0.001
	Yes	64 (5.8)	86 (7.8)	121 (11.0)	123 (11.2)	
CKD, *n* (%)	No	1,015 (92.2)	958 (87.1)	930 (84.6)	889 (80.8)	<0.001
	Yes	86 (7.8)	142 (12.9)	169 (15.4)	211 (19.2)	
Education, *n* (%)	Above high School	722 (65.6)	645 (58.6)	595 (54.1)	541 (49.2)	<0.001
	High School and below	379 (34.4)	455 (41.4)	504 (45.9)	559 (50.8)	
Marital Status, *n* (%)	Divorced	90 (8.2)	92 (8.4)	123 (11.2)	130 (11.8)	<0.001
	Living with partner	110 (10.0)	86 (7.8)	91 (8.3)	71 (6.5)	
	Married	500 (45.4)	560 (50.9)	539 (49.0)	579 (52.6)	
	Never married	348 (31.6)	267 (24.3)	209 (19.0)	157 (14.3)	
	Separated	31 (2.8)	30 (2.7)	32 (2.9)	49 (4.5)	
	Widowed	22 (2.0)	65 (5.9)	105 (9.6)	114 (10.4)	
INDFMPIR, *n* (%)	<1	196 (17.8)	224 (20.4)	202 (18.4)	220 (20.0)	0.125
	1.0–4.9	676 (61.4)	640 (58.2)	676 (61.5)	689 (62.6)	
	5+	229 (20.8)	236 (21.5)	221 (20.1)	191 (17.4)	
Smoking, *n* (%)	No	714 (64.9)	609 (55.4)	584 (53.1)	499 (45.4)	<0.001
	Yes	387 (35.1)	491 (44.6)	515 (46.9)	601 (54.6)	
Drinking, *n* (%)	No	763 (69.3)	818 (74.4)	779 (70.9)	835 (75.9)	0.001
	Yes	338 (30.7)	282 (25.6)	320 (29.1)	265 (24.1)	
Liver fibrosis, *n* (%)	No	875 (79.5)	770 (70.0)	714 (65.0)	659 (59.9)	<0.001
	Yes	226 (20.5)	330 (30.0)	385 (35.0)	441 (40.1)	
Death, *n* (%)	No	1,035 (94.0)	984 (89.5)	953 (86.7)	880 (80.0)	<0.001
	Yes	66 (6.0)	116 (10.5)	146 (13.3)	220 (20.0)	
SBP (mean, SD)		112.20 (15.44)	116.48 (17.50)	119.08 (19.25)	123.76 (19.69)	<0.001
DBP (mean, SD)		66.57 (10.37)	67.32 (10.48)	68.19 (11.42)	68.84 (12.50)	<0.001
Glucose (mean, SD)		90.94 (9.57)	95.37 (11.05)	98.97 (13.57)	117.01 (49.32)	<0.001
Triglycerides (mean, SD)		48.05 (10.29)	72.60 (10.62)	100.90 (16.40)	179.58 (78.33)	<0.001
AST (mean, SD)		24.08 (14.04)	24.23 (15.53)	25.01 (16.08)	25.35 (11.86)	0.117
ALT (mean, SD)		20.01 (11.30)	20.89 (15.50)	22.12 (18.89)	24.14 (15.34)	<0.001
Alb (mean, SD)		4.32 (0.33)	4.30 (0.34)	4.27 (0.35)	4.28 (0.36)	0.025
Globulin (mean, SD)		2.85 (0.47)	2.85 (0.43)	2.89 (0.45)	2.95 (0.48)	<0.001
TP (mean, SD)		7.16 (0.47)	7.15 (0.46)	7.17 (0.49)	7.23 (0.51)	<0.001
Chol (mean, SD)		173.92 (34.02)	186.38 (35.86)	197.89 (39.92)	209.02 (45.98)	<0.001
Creatitine (mean, SD)		0.83 (0.31)	0.88 (0.41)	0.90 (0.54)	0.92 (0.50)	<0.001
Tbil (mean, SD)		0.76 (0.32)	0.73 (0.32)	0.76 (0.34)	0.73 (0.34)	0.037
Triglyceride (mean, SD)		48.05 (10.29)	72.60 (10.62)	100.90 (16.40)	179.58 (78.33)	<0.001
Uric acid (mean, SD)		4.56 (1.09)	4.83 (1.20)	5.08 (1.27)	5.46 (1.37)	<0.001
APRI (mean, SD)		11.07 (9.68)	11.09 (10.61)	11.10 (9.44)	11.24 (7.51)	0.973
eGFR (mean, SD)		102.80 (27.73)	93.26 (28.62)	89.44 (28.83)	83.44 (26.57)	<0.001
LDH (mean, SD)		124.77 (27.45)	127.79 (30.64)	130.84 (32.02)	130.97 (28.71)	<0.001
GGT (mean, SD)		18.38 (19.08)	21.71 (30.02)	24.67 (35.74)	34.09 (49.08)	<0.001
Glucose (mean, SD)		90.94 (9.57)	95.37 (11.05)	98.97 (13.57)	117.01 (49.32)	<0.001
TyG (mean, SD)		7.66 (0.23)	8.13 (0.10)	8.49 (0.11)	9.14 (0.42)	<0.001
TyG-BMI (mean, SD)		168.87 (14.34)	181.13 (14.09)	191.74 (14.67)	209.91 (17.46)	<0.001
TyG-WC (mean, SD)		614.87 (55.56)	671.39 (60.64)	719.07 (61.97)	798.25 (77.23)	<0.001
TyG-WHtR (mean, SD)		3.67 (0.33)	4.01 (0.35)	4.29 (0.38)	4.78 (0.48)	<0.001
FIB-4 (mean, SD)		0.99 (0.91)	1.15 (0.90)	1.22 (0.85)	1.27 (0.75)	<0.001
WC (mean, SD)		80.27 (6.62)	82.54 (7.27)	84.65 (7.14)	87.31 (7.05)	<0.001

CVD, cardiovascular disease; BMI, body mass index; SBP, systolic blood pressure; DBP, diastolic blood pressure; AST, aspartate aminotransferase; ALT, alanine aminotransferase; Tbil, total bilirubin; GGT, gamma-glutamyl transferase; ALB, albumin; TP, total protein; Chol, cholesterol; TG, triglycerides; TyG, triglyceride-glucose index; TyG-BMI, TyG-body mass index; TyG-WC, TyG-waist circumference; TyG-WHtR, TyG-waist-to-height ratio; FIB-4, fibrosis-4 score; APRI, aspartate transaminase-to-platelet ratio index; eGFR, estimated glomerular filtration rate; WC, waist circumference.

### Linear regression analysis about the relationship between TyG, TyG-BMI, TyG-Wc, TyG-WHtR and liver function metrics, kidney function metrics, and blood lipids

**Figure 1:**
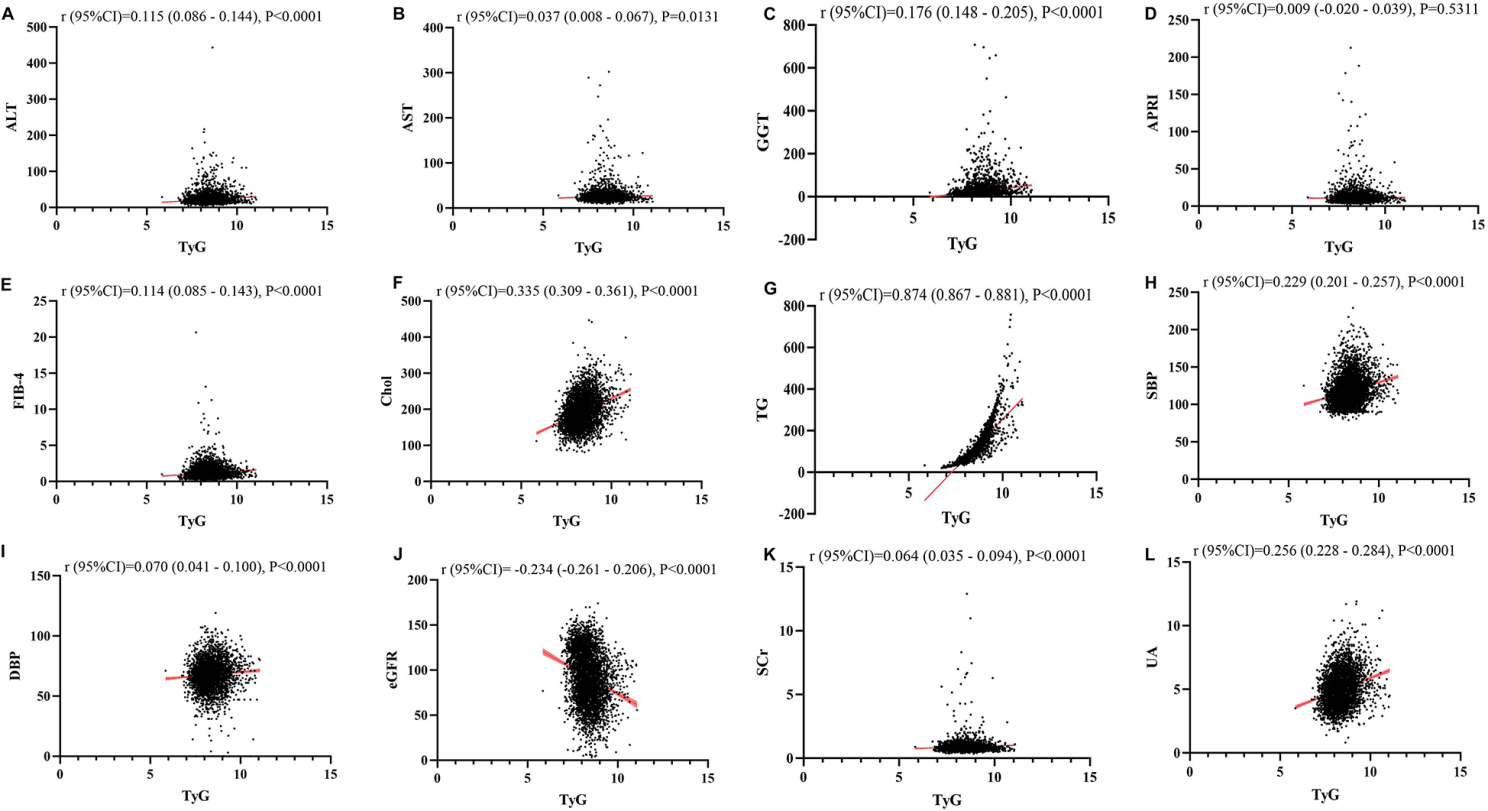
**(A–L)** Linear correlation between TyG index and biochemical parameters. Relationship between TyG and liver function metrics [**(A)** ALT; **(B)** AST; **(C)** GGT; **(D)** FIB-4; **(E)** APRI], blood lipids [**(F)** Chol; **(G)** TG], blood pressure [**(H)** SBP; **(I)** DBP] and kidney function metrics [**(J)** eGFR; **(K)** Scr; **(L)** UA]. ALT, alanine aminotransferase; AST, aspartate aminotransferase; GGT, gammaglutamyl transferase; APRI, aspartate transaminase-to-platelet ratio index; FIB-4, fibrosis-4 score; Chol, cholesterol; TG, triglycerides; SBP, systolic blood pressure; DBP, diastolic blood pressure; eGFR, estimated glomerular filtration rate; Scr, serum creatinine; UA, uric acid.

**Figure 2:**
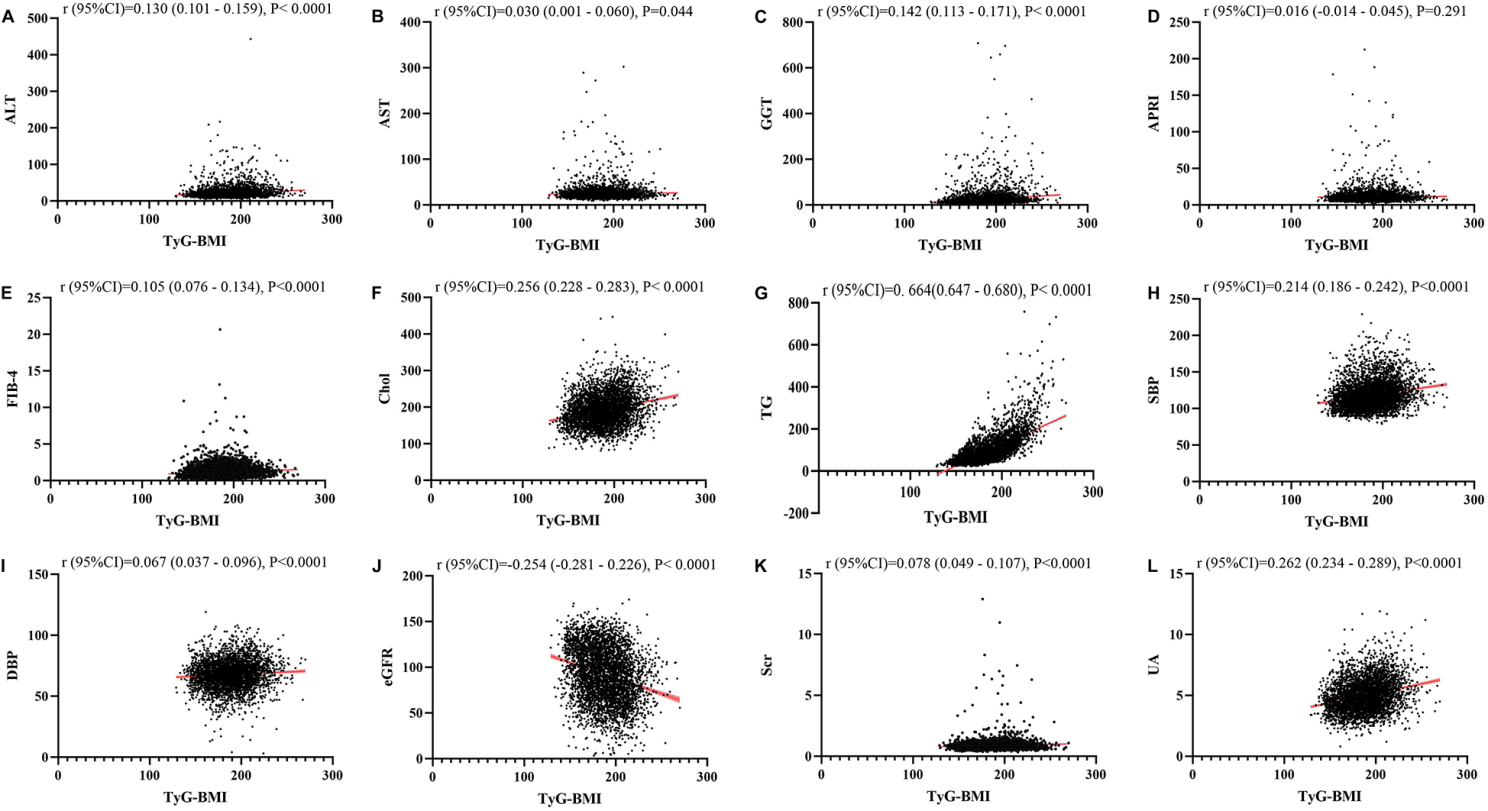
**(A–L)** Linear correlation between TyG-BMI index and biochemical parameters. Relationship between TyG and liver function metrics [**(A)** ALT; **(B)** AST; **(C)** GGT; **(D)** FIB-4; **(E)** APRI], blood lipids [**(F)** Chol; **(G)** TG], blood pressure [**(H)** SBP; **(I)** DBP] and kidney function metrics [**(J)** eGFR; **(K)** Scr; **(L)** UA]. ALT, alanine aminotransferase; AST, aspartate aminotransferase; GGT, gammaglutamyl transferase; APRI, aspartate transaminase-to-platelet ratio index; FIB-4, fibrosis-4 score; Chol, cholesterol; TG, triglycerides; SBP, systolic blood pressure; DBP, diastolic blood pressure; eGFR, estimated glomerular filtration rate; Scr, serum creatinine; UA, uric acid.

**Figure 3:**
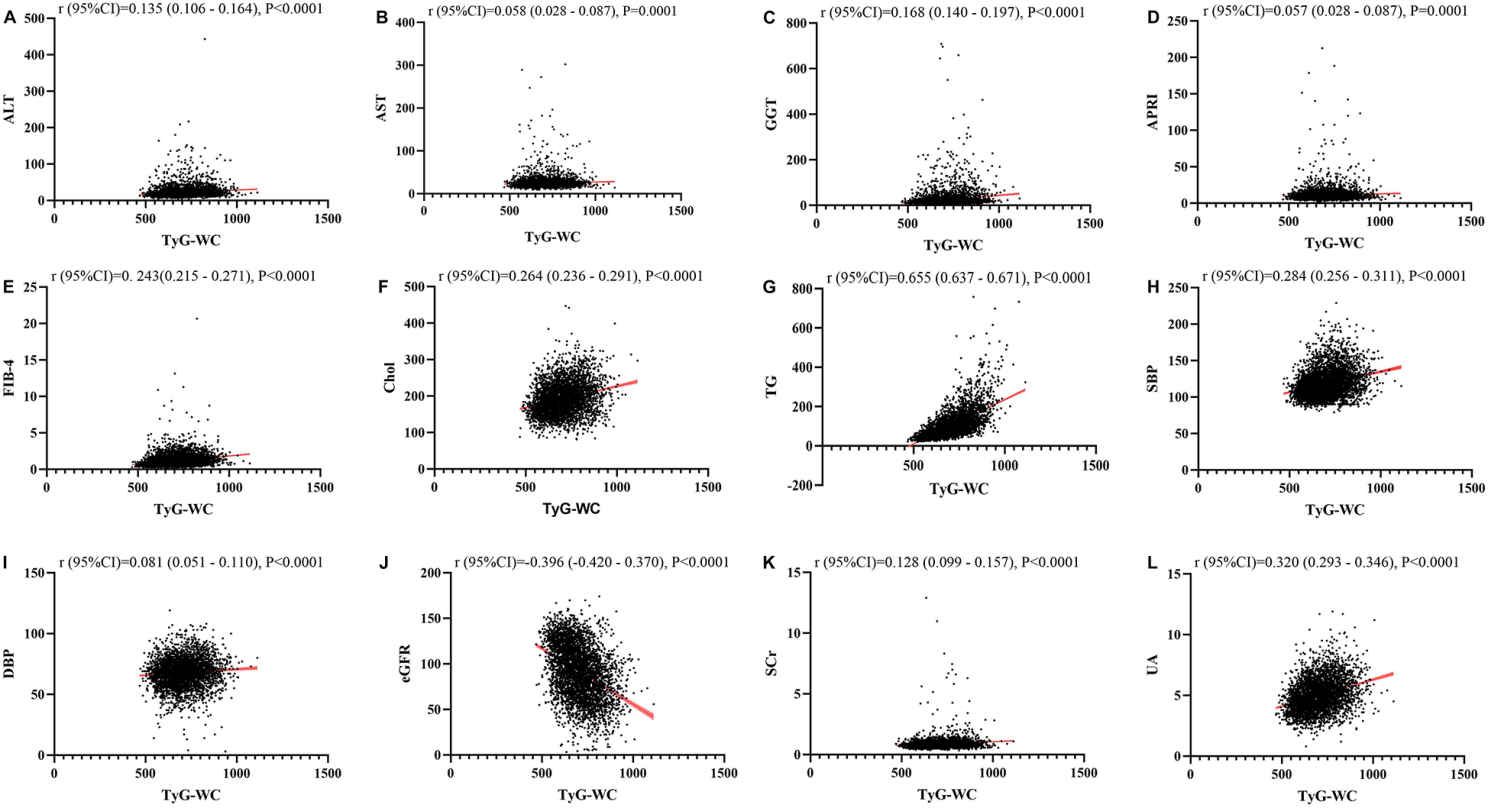
**(A–L)** Linear correlation between TyG-WC index and biochemical parameters. Relationship between TyG and liver function metrics [**(A)** ALT; **(B)** AST; **(C)** GGT; **(D)** FIB-4; **(E)** APRI], blood lipids [**(F)** Chol; **(G)** TG], blood pressure [**(H)** SBP; **(I)** DBP] and kidney function metrics [**(J)** eGFR; **(K)** Scr; **(L)** UA]. ALT, alanine aminotransferase; AST, aspartate aminotransferase; GGT, gammaglutamyl transferase; APRI, aspartate transaminase-to-platelet ratio index; FIB-4, fibrosis-4 score; Chol, cholesterol; TG, triglycerides; SBP, systolic blood pressure; DBP, diastolic blood pressure; eGFR, estimated glomerular filtration rate; Scr, serum creatinine; UA, uric acid.

**Figure 4:**
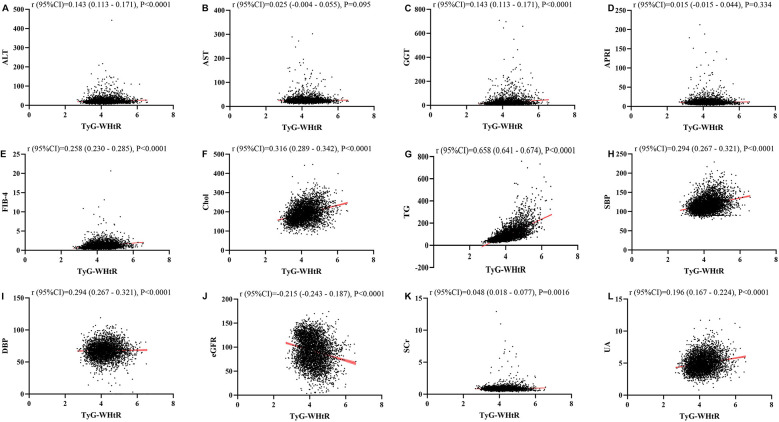
**(A–L)** Linear correlation between TyG-wHtR index and biochemical parameters. Relationship between TyG and liver function metrics [**(A)** ALT; **(B)** AST; **(C)** GGT; **(D)** FIB-4; **(E)** APRI], blood lipids [**(F)** Chol; **(G)** TG], blood pressure [**(H)** SBP; **(I)** DBP] and kidney function metrics [**(J)** eGFR; **(K)** Scr; **(L)** UA]. ALT, alanine aminotransferase; AST, aspartate aminotransferase; GGT, gammaglutamyl transferase; APRI, aspartate transaminase-to-platelet ratio index; FIB-4, fibrosis-4 score; Chol, cholesterol; TG, triglycerides; SBP, systolic blood pressure; DBP, diastolic blood pressure; eGFR, estimated glomerular filtration rate; Scr, serum creatinine; UA, uric acid.

Figures 1–4 illustrated the association between TyG and its composite indicators of obesity with liver function metrics (ALT, AST, GGT, FIB-4, APRI), kidney function metrics (creatinine, eGFR, uric acid), and blood lipids (triglycerides, cholesterol). Through linear regression analysis, it was found that as TyG, TyG-BMI, TyG-WC, and TyG-WHtR increasing, ALT, AST, GGT, FIB-4, APRI, creatinine, uric acid, triglycerides, and cholesterol increased, while eGFR decreased (*P* < 0.05).

### The relationship between TyG, TyG-Bm, TyG-Wc, and TyG-WHtR and liver fibrosis, CVD, stroke, and death

**Figure 5:**
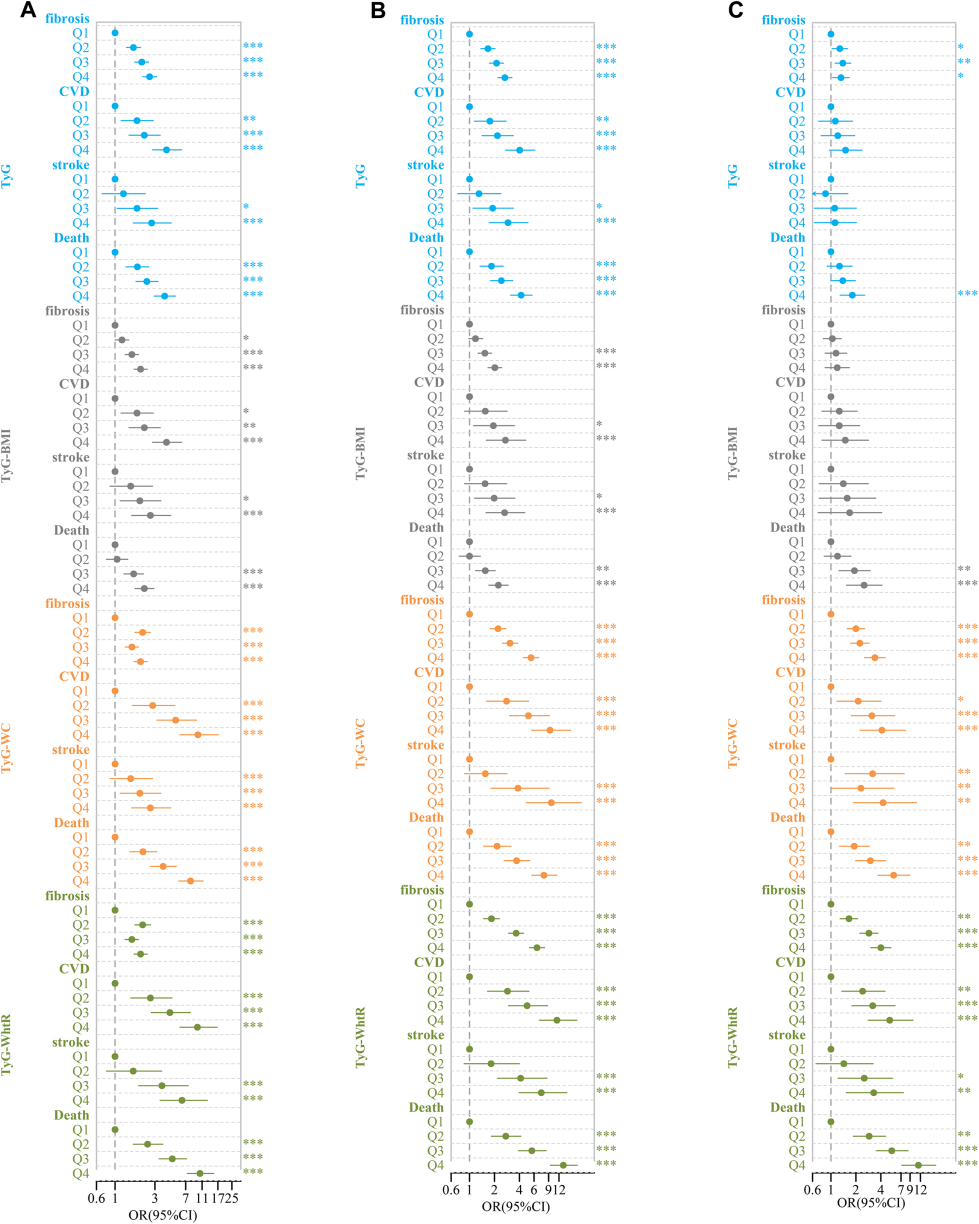
Forest plot of TyG-related indices and adverse clinical outcomes. **(A)** Rude model without adjustment; **(B)** adjusted for age, gender, race; **(C)** further adjusted for BMI, hypertension, diabetes, education level, marital status, ratio of family income to poverty, smoking, and drinking.*P < 0.05, **P < 0.01, ***P < 0.001.

After conducting univariate logistic and Cox regression analyses, it was observed that TyG had a significant correlation with liver fibrosis, CVD, stroke, and death. Compared to the lowest quartile (Q1), the OR and 95% CI for Q4 were 2.59 (2.15, 3.14), 4.13 (2.81, 6.25), 2.74 (1.65, 4.71), and 3.92 (2.95, 5.27), respectively. However, after controlling for variables such as age, gender, race, BMI, hypertension, diabetes, education level, marital status, family income-to-poverty ratio, smoking, and alcohol consumption, the OR and 95% CI for Q4 compared to Q1 were 0.89 (0.64, 1.24), 1.14 (0.66, 2.04), 0.85 (0.43, 1.76), and 1.19 (0.78, 1.85), respectively (Figure 5).

Similarly, in univariate logistic and Cox regression analyses, TyG-BMI was significantly associated with liver fibrosis, CVD, stroke, and death. Compared to Q1, the OR and 95% CI for Q4 were 2.02 (1.69, 2.43), 4.13 (2.81, 6.25), 2.65 (1.58, 4.64), and 2.24 (1.73, 2.92), respectively. After controlling for the same covariates, the OR and 95%CI for Q4 compared to Q1 were 0.75 (0.46, 1.23), 1.33 (0.57, 3.12), 1.16 (0.39, 3.51), and 1.16 (0.39, 3.51), respectively (Figure 5).

In the analysis with TyG-WC, we found similar significant associations with liver fibrosis, CVD, stroke, and death. Compared to Q1, the OR and 95% CI for Q4 were 2.02 (1.69, 2.43), 9.8 (5.98, 17.2), 2.65 (1.58, 4.64), and 8.00 (5.79, 11.31), respectively. After accounting for the covariates, the OR and 95%CI for Q4 in comparison to Q1 were 2.54 (1.68, 3.87), 3.28 (1.52, 7.56), 3.29 (1.22, 10.04), and 3.18 (1.83, 5.61), respectively (Figure 5).

Finally, for TyG-WHtR, significant associations with liver fibrosis, CVD, stroke, and death were observed. Compared to Q1, the OR and 95% CI for Q4 were 2.02 (1.69, 2.43), 9.67 (5.98, 16.69), 6.32 (3.46, 12.71), and 10.39 (7.33, 15.18), respectively. After adjusting for the same set of covariates, the OR and 95% CI for Q4 compared to Q1 were 3.17 (2.12, 4.77), 4.35 (2.05, 9.81), 2.35 (0.96, 6.18), and 6.03 (3.47, 10.72), respectively (Figure 5).

### The survival analysis of the TyG, TyG-BMI, TyG-WC, and TyG-WHtR

**Figure 6:**
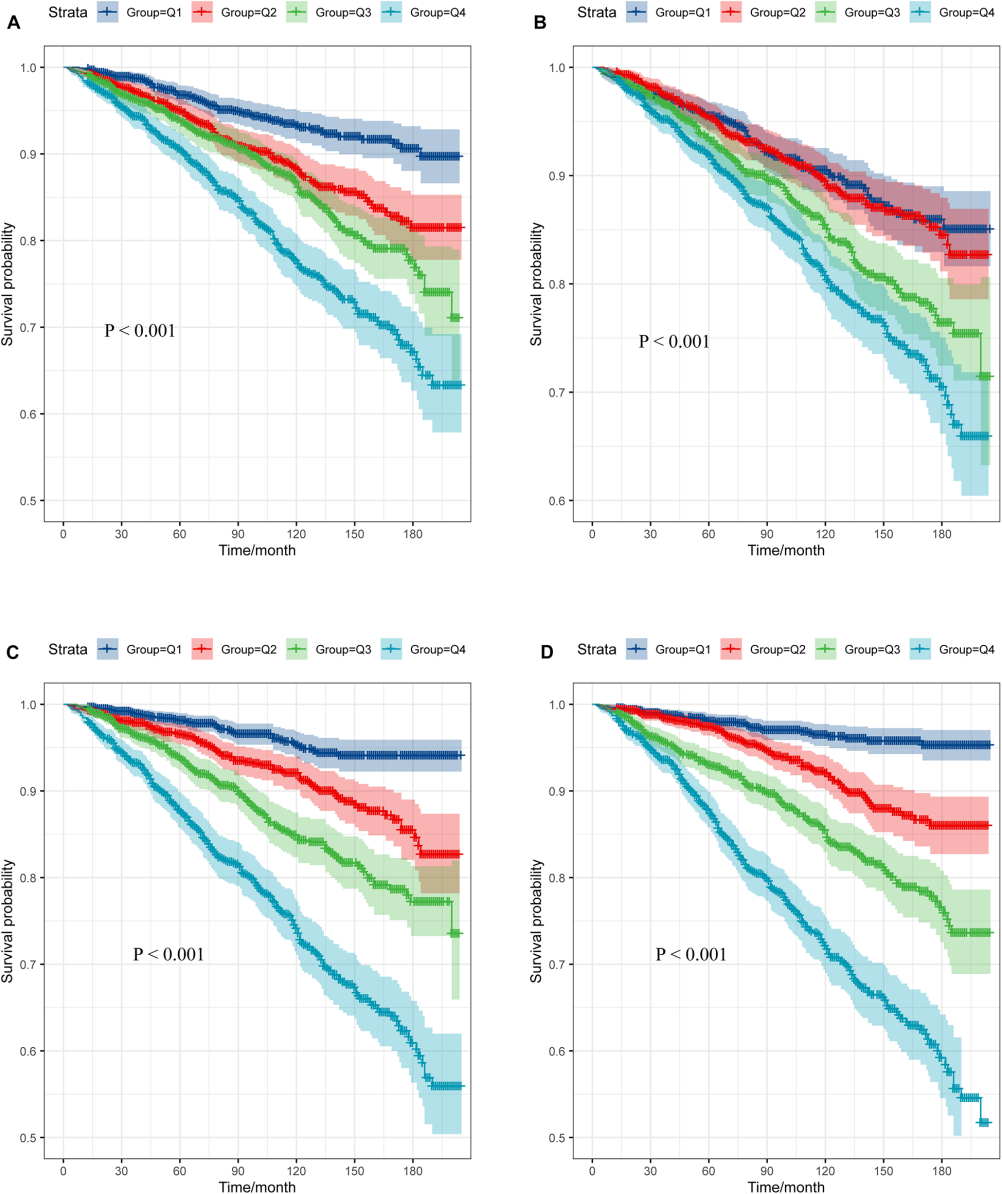
Survival curve of subjects across different TyG-related indices quartile. **(A)** TyG; **(B)** TyG-BMI; **(C)** TyG-WC; **(D)** TyG-WHtR.

The participants were classified into four groups (Q1, Q2, Q3, Q4) according to the quartiles of TyG, TyG-BMI, TyG-WC, and TyG-WHtR, with the Q1 group acting as the reference category. The study showed that as TyG, TyG-WC, TyG-WHtR, TyG-BMI index increases, the survival rate of the study population decreases, which is statistically significant (*P* < 0.001) (Figure 6).

### Subgroup analysis

A stratified analysis was conducted according to gender, smoking, education level, hypertension, diabetes, and CVD. Subgroup analysis revealed that the association between TyG-related indices and adverse clinical outcomes was generally consistent across the majority of subgroups. Furthermore, because of the small sample size of patients with hypertension, the subgroup analysis was performed exclusively on the group without hypertension (Supplementary Figures S1–S11).

## Discussion

This study included 4,440 individuals aged over 20 years with a BMI less than 25 from the NHANES 2003–2018 cycle. We analyzed the association between different insulin resistance indicators—TyG, TyG-BMI, TyG-WC, and TyG-WHtR—with the risk of adverse clinical outcomes. After adjusting for confounding factors, TyG-WC, and TyG-WHtR were found to be significantly associated with liver fibrosis, CVD, stroke, and all-cause mortality. These results highlighted their potential clinical value in identifying individuals at risk for metabolic and cardiovascular diseases, even in populations considered to have normal weight.

The TyG index, calculated from triglyceride and glucose levels, has gained widespread recognition as a reliable indicator of insulin resistance (12, 13). When comparing the predictive power of TyG, TyG-BMI, TyG-WC, and TyG-WHtR, our results indicated some notable differences. While all four indices were associated with adverse clinical outcomes, and TyG-WC and TyG-WHtR showed the strongest associations. This is consistent with the growing recognition that abdominal obesity, as indicated by waist circumference and waist-to-height ratio, is essential in the progression of insulin resistance and related disorders (22–24). Visceral fat is known to increase triglyceride production (25), impair insulin action, and promote systemic inflammation (26–28), all of which play a role in the onset of cardiovascular disease and various metabolic conditions. And visceral fat is metabolically active and more closely linked to insulin resistance and associated diseases than subcutaneous fat (29, 30). In addition, numerous studies have demonstrated that waist circumference and waist-to-height ratio outperform BMI in predicting metabolic risk, especially in individuals with normal weight, as they more accurately represent fat distribution in the body (31–34). The inclusion of BMI in the TyG-BMI model may be less informative in predicting the risk of adverse outcomes in individuals with normal weight, as BMI alone does not distinguish between lean mass and fat mass or provide insights into fat distribution.

The relationship between TyG and insulin resistance is rooted in the pathophysiology of lipid and glucose metabolism. Elevated triglyceride levels reflect impaired lipoprotein lipase activity, a key enzyme in triglyceride metabolism, which is often seen in insulin-resistant individuals (35, 36). In addition, higher glucose concentrations are a direct consequence of insulin's diminished ability to regulate blood sugar, leading to hyperglycemia. Both metabolic abnormalities are characteristic of insulin resistance and play a role in the progression of metabolic diseases. The TyG index captures this dysregulation, making it a valuable marker for identifying individuals at risk for these diseases.

In the context of individuals with normal BMI, our study showed that insulin resistance is a significant concern in this population. While BMI remains a widely used metric for assessing obesity and metabolic risk, it fails to capture the full spectrum of metabolic abnormalities (37). Our findings underscore the importance of utilizing more comprehensive TyG-WC and TyG-WHtR in the assessment of adverse clinical outcomes in normal-weight individuals. These markers provide a clearer picture of metabolic health, allowing for earlier intervention and better risk stratification for adverse clinical outcomes.

The study also has certain limitations. The study was limited by a small sample size, which resulted in a wide confidence interval when assessing statistical efficiency. This reduced the precision of the findings and may have hindered the ability to identify true significant effects, particularly in the subgroup analysis, due to the small sample size in subgroups such as those with diabetes and hypertension. Secondly, the sample was predominantly composed of non-Hispanic White individuals, which could constrain the ability to extrapolate the results to other populations. Third, emerging evidence suggested that insulin resistance plays a pivotal role in the pathophysiology of heart failure with preserved ejection fraction (HFpEF) (38), even in non-obese individuals, the lack of left ventricular ejection fraction assessment precluded the differentiation of HFpEF. Fourth, our study relied on non-invasive liver fibrosis measures, which have inherent variability and limited accuracy.

## Conclusion

This study utilized the NHANES database to examine how insulin resistance-related parameters, including TyG, TyG-BMI, TyG-WC, and TyG-WHtR, predict the risk of adverse clinical outcomes in individuals with a normal BMI. The results revealed that TyG-WC and TyG-WHtR were strongly linked to an increased risk of liver fibrosis, cardiovascular disease, and all-cause mortality, highlighting that insulin resistance is common even among those with a normal BMI. Moreover, TyG-WC and TyG-WHtR could serve as potential indicators of insulin resistance in this group.

## Data Availability

The datasets presented in this study can be found in online repositories. The names of the repository/repositories and accession number(s) can be found in the article/Supplementary Material.

## Supplementary material

Supplementary Figure 1 Forest plot of TyG-related indices and adverse clinical outcomes in female, after adjusting for age, gender, race, BMI, hypertension, diabetes, education level, marital status, ratio of family income to poverty, smoking, and drinking. **P* < 0.05, ***P* < 0.01, ****P* < 0.001.

Supplementary Figure 2 Forest plot of TyG-related indices and adverse clinical outcomes in male, after adjusting for age, gender, race, BMI, hypertension, diabetes, education level, marital status, ratio of family income to poverty, smoking, and drinking. **P* < 0.05, ***P* < 0.01, ****P* < 0.001.

Supplementary Figure 3 Forest plot of TyG-related indices and adverse clinical outcomes in subjects with diabetes, after adjusting for age, gender, race, BMI, hypertension, diabetes, education level, marital status, ratio of family income to poverty, smoking, and drinking. **P* < 0.05, ***P* < 0.01, ****P* < 0.001.

Supplementary Figure 4 Forest plot of TyG-related indices and adverse clinical outcomes in subjects without diabetes, after adjusting for age, gender, race, BMI, hypertension, diabetes, education level, marital status, ratio of family income to poverty, smoking, and drinking. **P* < 0.05, ***P* < 0.01, ****P* < 0.001.

Supplementary Figure 5 Forest plot of TyG-related indices and adverse clinical outcomes in subjects with CKD, after adjusting for age, gender, race, BMI, hypertension, diabetes, education level, marital status, ratio of family income to poverty, smoking, and drinking. **P* < 0.05, ***P* < 0.01, ****P* < 0.001.

Supplementary Figure 6 Forest plot of TyG-related indices and adverse clinical outcomes in subjects without CKD, after adjusting for age, gender, race, BMI, hypertension, diabetes, education level, marital status, ratio of family income to poverty, smoking, and drinking. **P* < 0.05, ***P* < 0.01, ****P* < 0.001.

Supplementary Figure 7 Forest plot of TyG-related indices and adverse clinical outcomes in subjects with smoking, after adjusting for age, gender, race, BMI, hypertension, diabetes, education level, marital status, ratio of family income to poverty, smoking, and drinking. **P* < 0.05, ***P* < 0.01, ****P* < 0.001.

Supplementary Figure 8 Forest plot of TyG-related indices and adverse clinical outcomes in subjects without smoking, after adjusting for age, gender, race, BMI, hypertension, diabetes, education level, marital status, ratio of family income to poverty, smoking, and drinking. **P* < 0.05, ***P* < 0.01, ****P* < 0.001.

Supplementary Figure 9 Forest plot of TyG-related indices and adverse clinical outcomes in subjects with high school and below degree, after adjusting for age, gender, race, BMI, hypertension, diabetes, education level, marital status, ratio of family income to poverty, smoking, and drinking. **P* < 0.05, ***P* < 0.01, ****P* < 0.001.

Supplementary Figure 10 Forest plot of TyG-related indices and adverse clinical outcomes in subjects with above high school degree, after adjusting for age, gender, race, BMI, hypertension, diabetes, education level, marital status, ratio of family income to poverty, smoking, and drinking. **P* < 0.05, ***P* < 0.01, ****P* < 0.001.

Supplementary Figure 11 Forest plot of TyG-related indices and adverse clinical outcomes in subjects without hypertension, after adjusting for age, gender, race, BMI, hypertension, diabetes, education level, marital status, ratio of family income to poverty, smoking, and drinking. **P* < 0.05, ***P* < 0.01, ****P* < 0.001.
