# Supplementary figures and images for: Association of triglyceride-glucose-related indices with adverse clinical outcomes in individuals with normal body mass index
Source: Front Cardiovasc Med. 2025 Apr 24;12:1570239. doi: 10.3389/fcvm.2025.1570239 (PMC12058858; doi:10.3389/fcvm.2025.1570239)

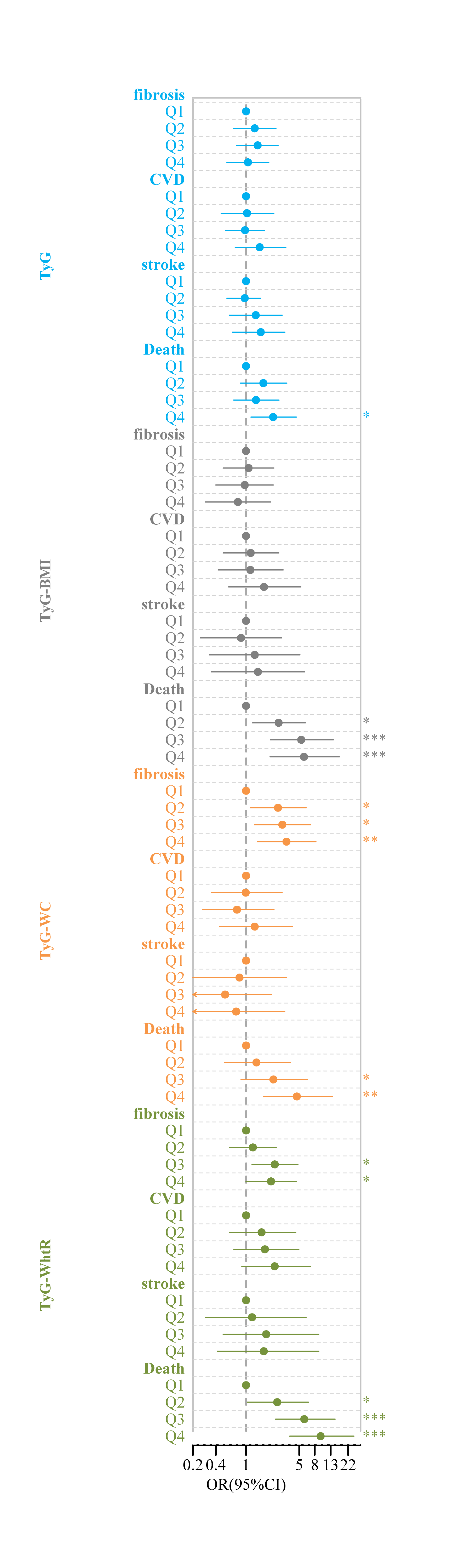

Supplement: Supplementary Figure 1 — Forest plot of TyG-related indices and adverse clinical outcomes in female, after adjusting for age, gender, race, BMI, hypertension, diabetes, education level, marital status, ratio of family income to poverty, smoking, and drinking. *P < 0.05, **P < 0.01, ***P < 0.001. [file Image1.tif]

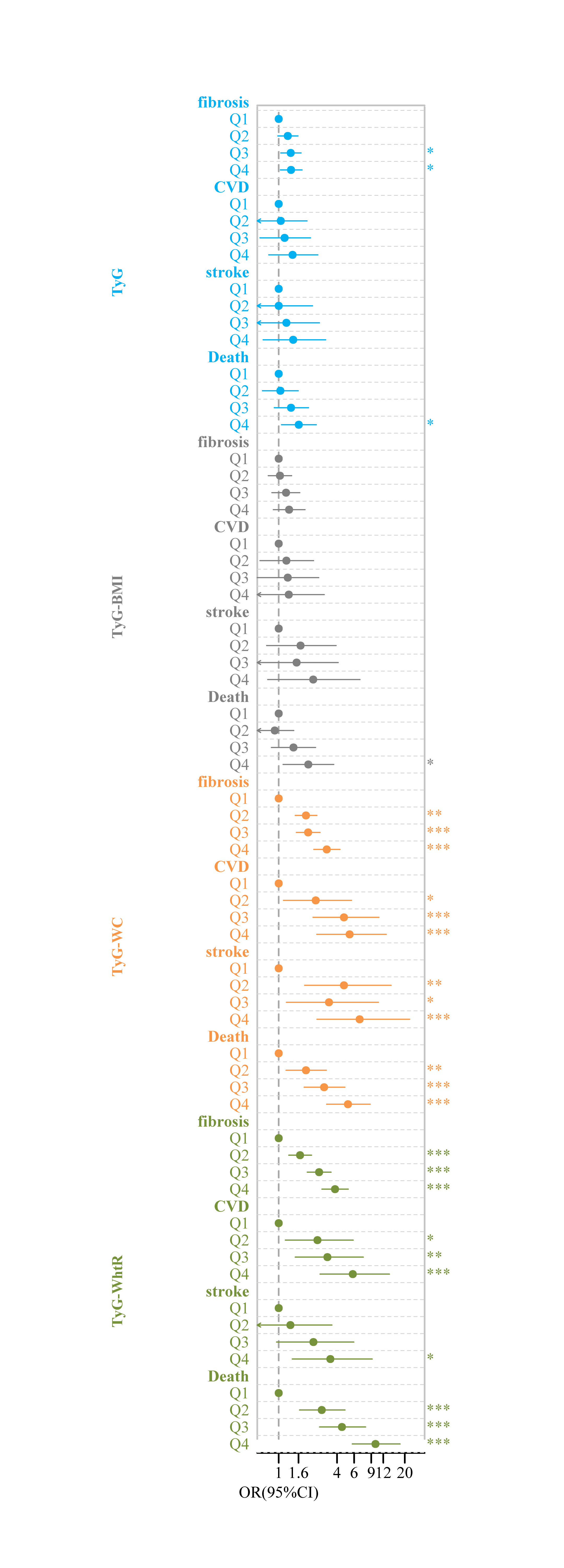

Supplement: Supplementary Figure 2 — Forest plot of TyG-related indices and adverse clinical outcomes in male, after adjusting for age, gender, race, BMI, hypertension, diabetes, education level, marital status, ratio of family income to poverty, smoking, and drinking. *P < 0.05, **P < 0.01, ***P < 0.001. [file Image2.tif]

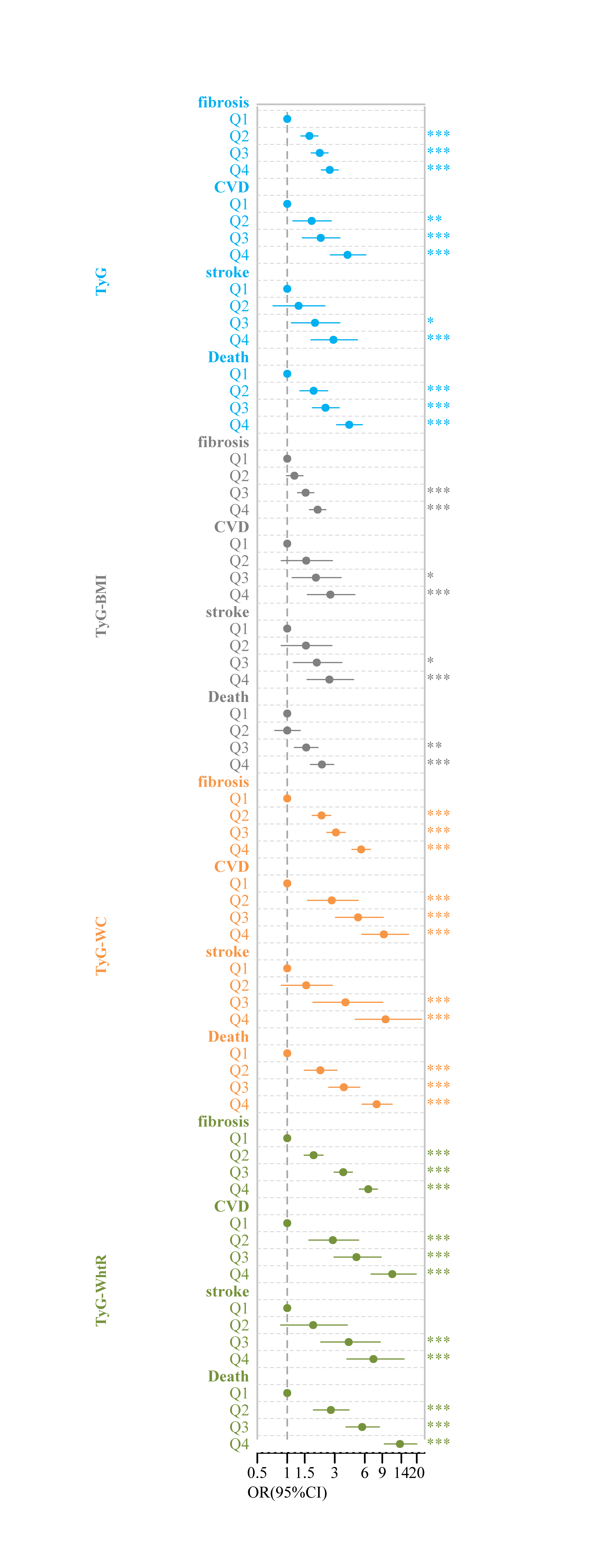

Supplement: Supplementary Figure 3 — Forest plot of TyG-related indices and adverse clinical outcomes in subjects with diabetes, after adjusting for age, gender, race, BMI, hypertension, diabetes, education level, marital status, ratio of family income to poverty, smoking, and drinking. *P < 0.05, **P < 0.01, ***P < 0.001. [file Image3.tif]

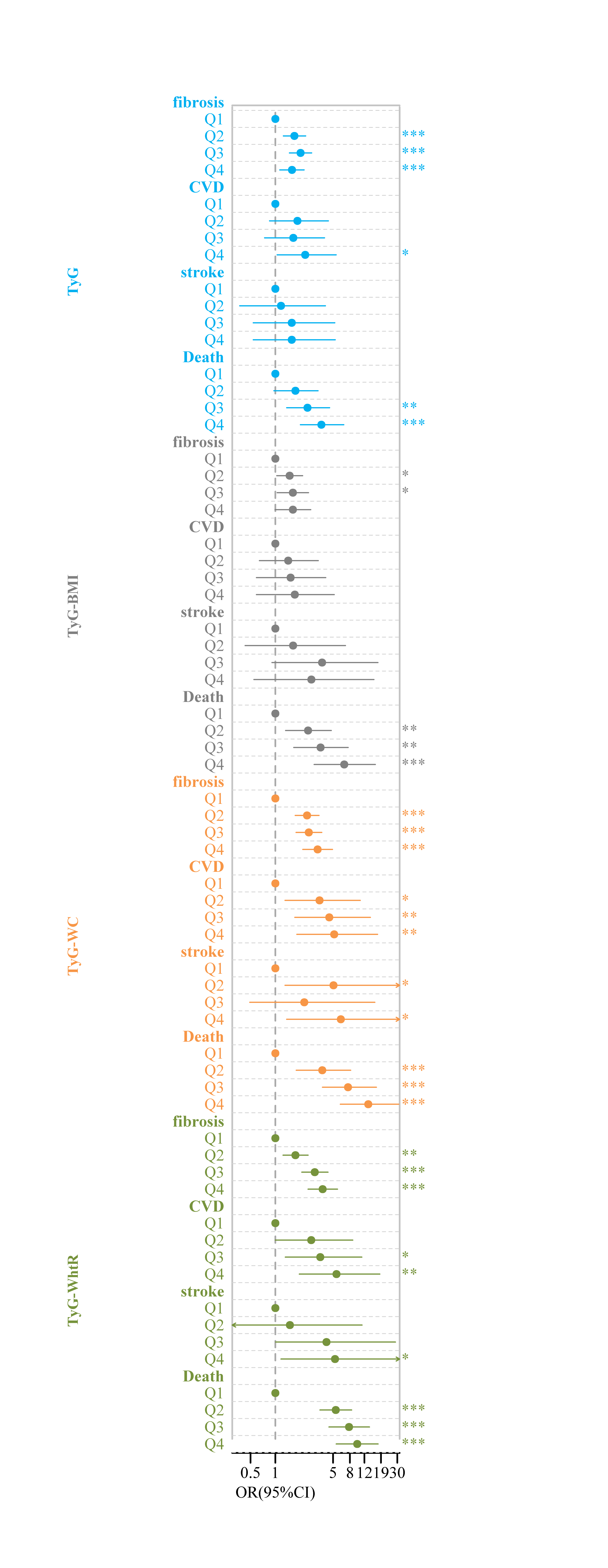

Supplement: Supplementary Figure 4 — Forest plot of TyG-related indices and adverse clinical outcomes in subjects without diabetes, after adjusting for age, gender, race, BMI, hypertension, diabetes, education level, marital status, ratio of family income to poverty, smoking, and drinking. *P < 0.05, **P < 0.01, ***P < 0.001. [file Image4.tif]

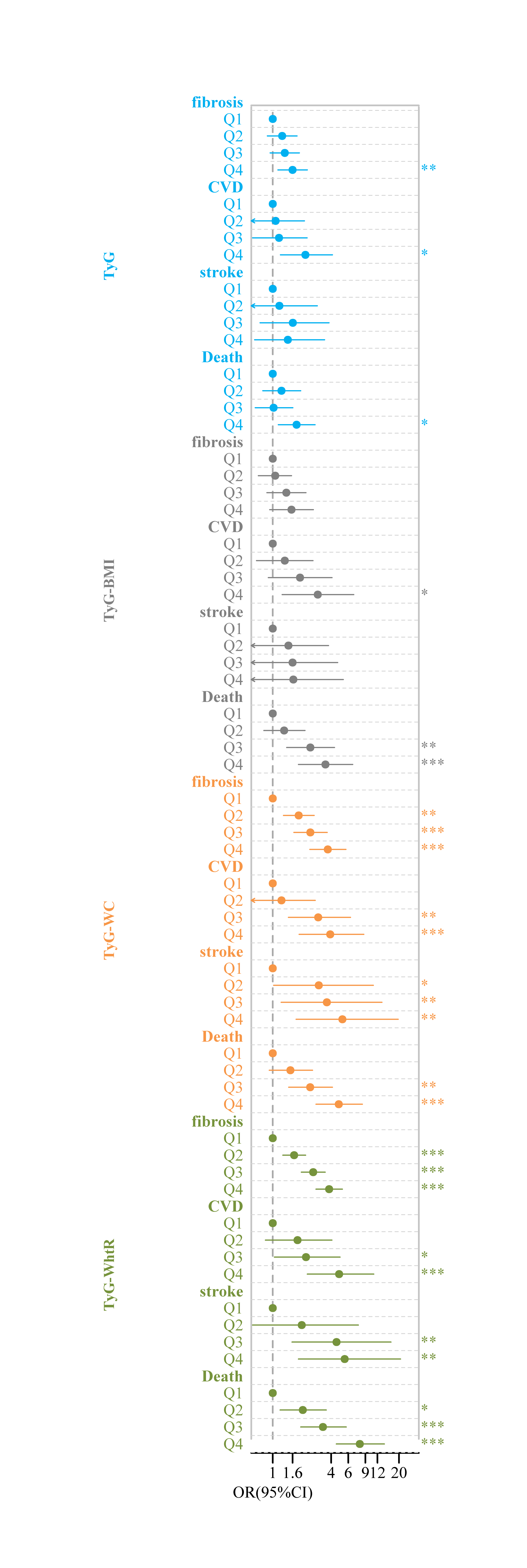

Supplement: Supplementary Figure 5 — Forest plot of TyG-related indices and adverse clinical outcomes in subjects with CKD, after adjusting for age, gender, race, BMI, hypertension, diabetes, education level, marital status, ratio of family income to poverty, smoking, and drinking. *P < 0.05, **P < 0.01, ***P < 0.001. [file Image5.tif]

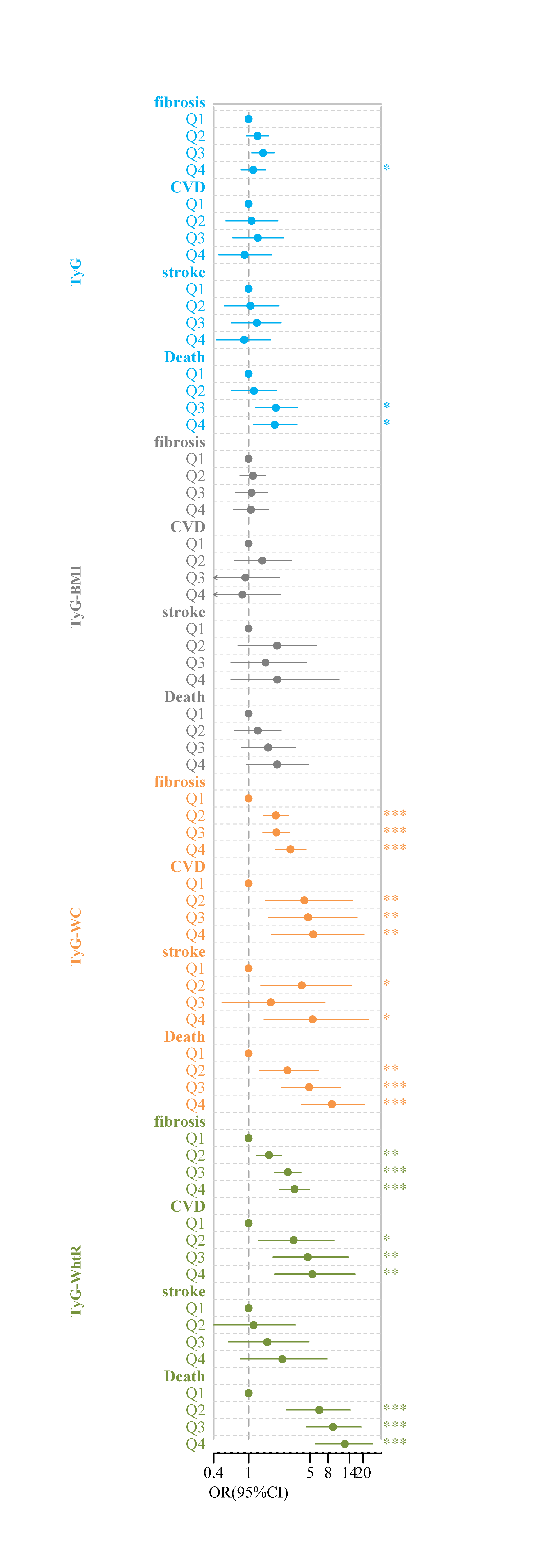

Supplement: Supplementary Figure 6 — Forest plot of TyG-related indices and adverse clinical outcomes in subjects without CKD, after adjusting for age, gender, race, BMI, hypertension, diabetes, education level, marital status, ratio of family income to poverty, smoking, and drinking. *P < 0.05, **P < 0.01, ***P < 0.001. [file Image6.tif]

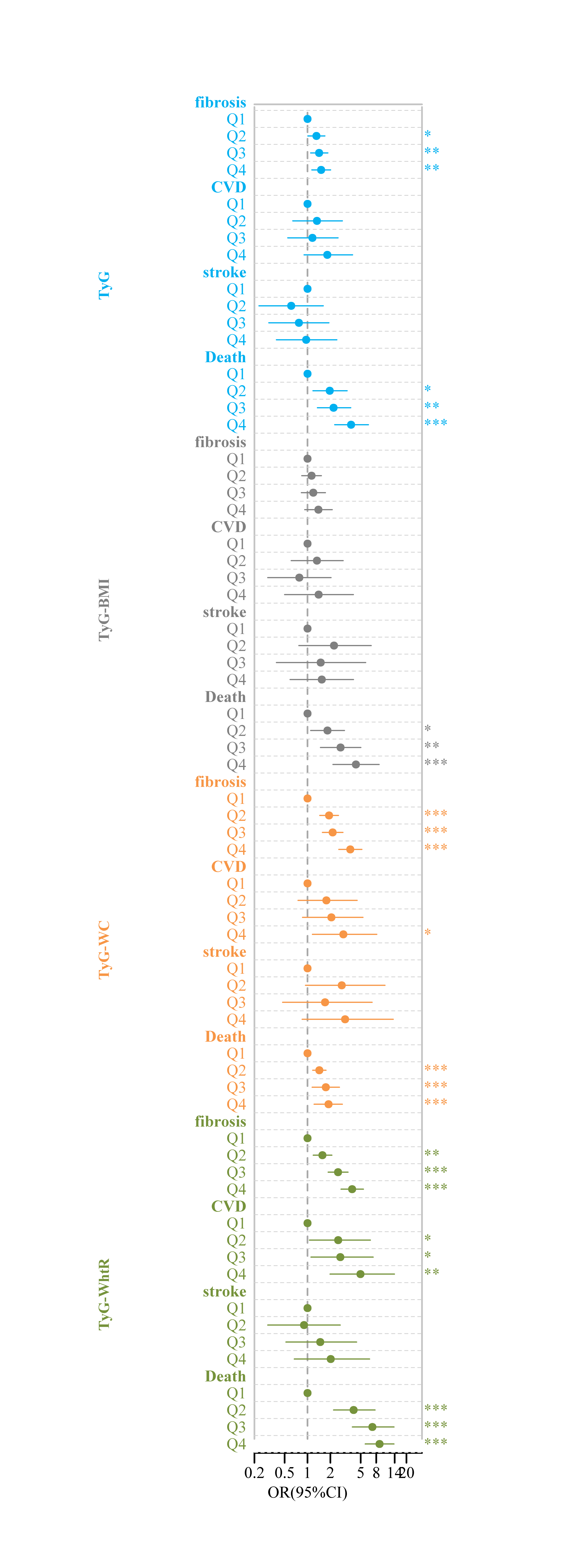

Supplement: Supplementary Figure 7 — Forest plot of TyG-related indices and adverse clinical outcomes in subjects with smoking, after adjusting for age, gender, race, BMI, hypertension, diabetes, education level, marital status, ratio of family income to poverty, smoking, and drinking. *P < 0.05, **P < 0.01, ***P < 0.001. [file Image7.tif]

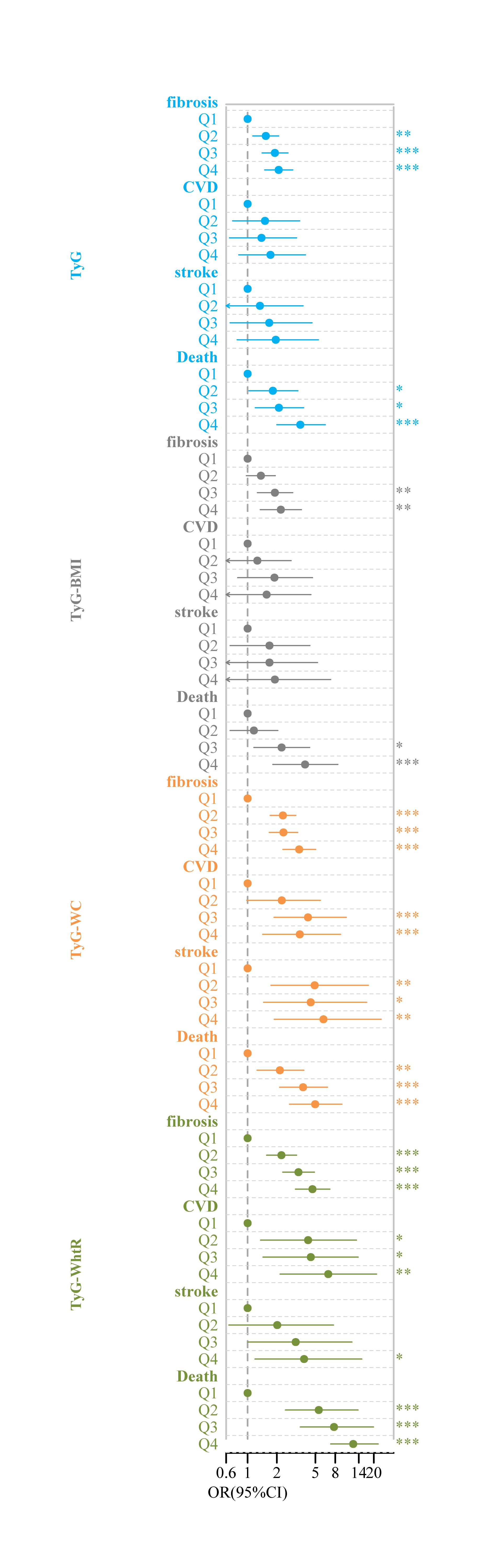

Supplement: Supplementary Figure 8 — Forest plot of TyG-related indices and adverse clinical outcomes in subjects without smoking, after adjusting for age, gender, race, BMI, hypertension, diabetes, education level, marital status, ratio of family income to poverty, smoking, and drinking. *P < 0.05, **P < 0.01, ***P < 0.001. [file Image8.tif]

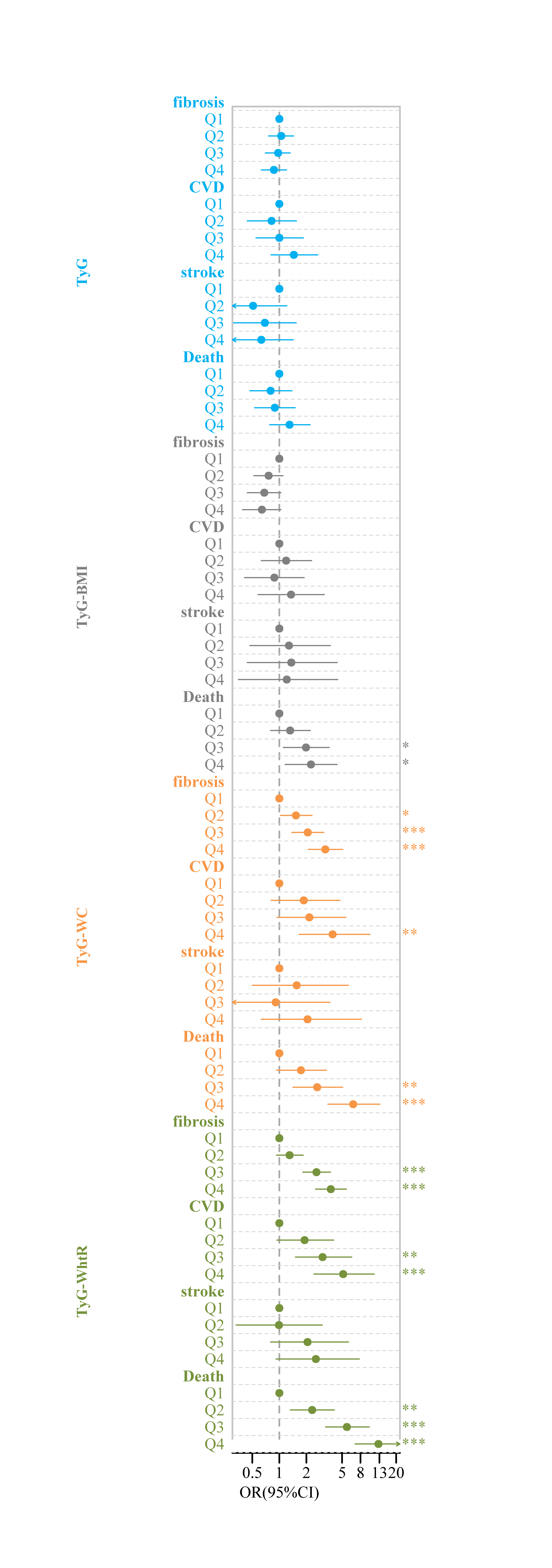

Supplement: Supplementary Figure 9 — Forest plot of TyG-related indices and adverse clinical outcomes in subjects with high school and below degree, after adjusting for age, gender, race, BMI, hypertension, diabetes, education level, marital status, ratio of family income to poverty, smoking, and drinking. *P < 0.05, **P < 0.01, ***P < 0.001. [file Image9.tif]

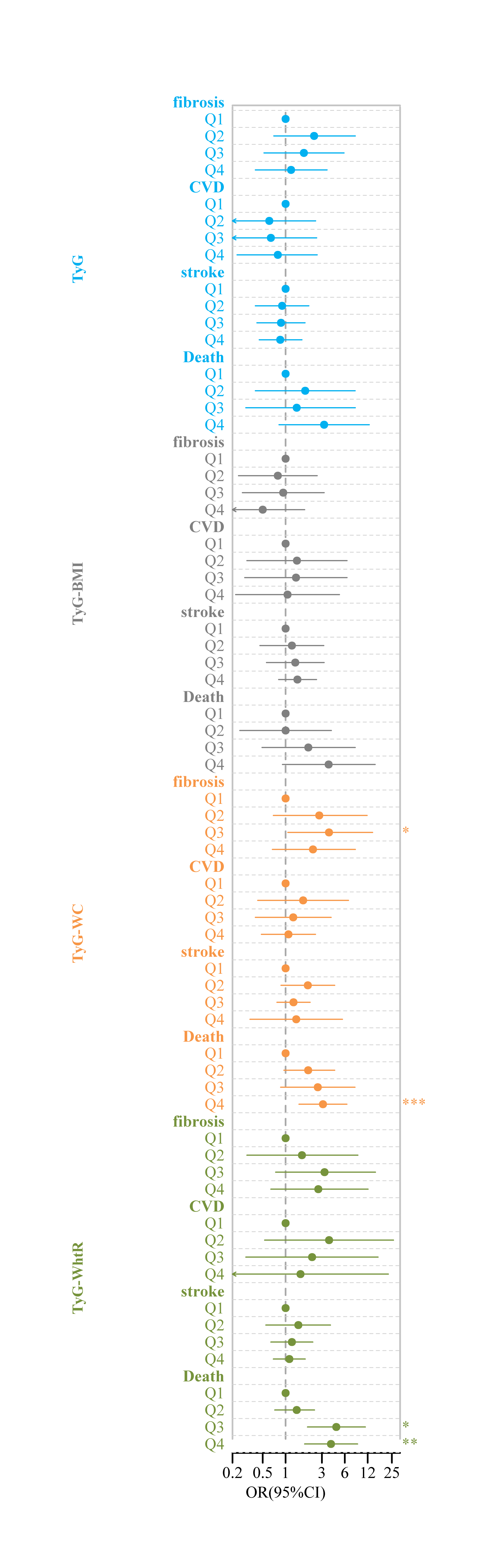

Supplement: Supplementary Figure 10 — Forest plot of TyG-related indices and adverse clinical outcomes in subjects with above high school degree, after adjusting for age, gender, race, BMI, hypertension, diabetes, education level, marital status, ratio of family income to poverty, smoking, and drinking. *P < 0.05, **P < 0.01, ***P < 0.001. [file Image10.tif]

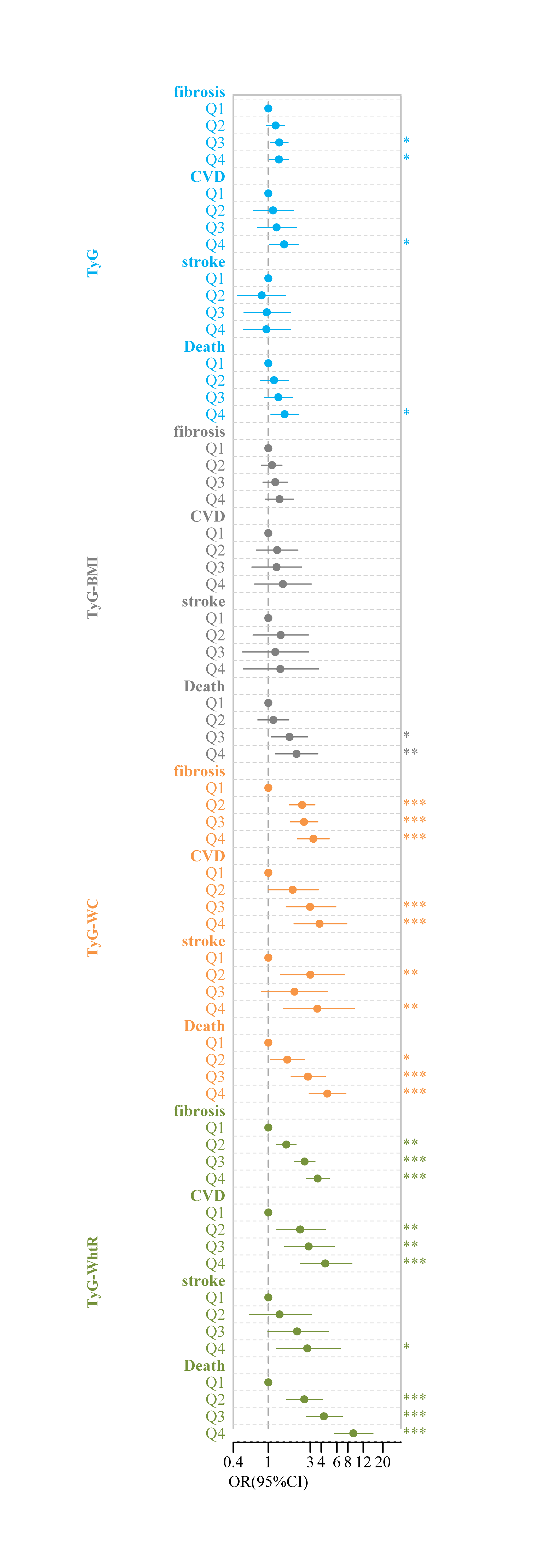

Supplement: Supplementary Figure 11 — Forest plot of TyG-related indices and adverse clinical outcomes in subjects without hypertension, after adjusting for age, gender, race, BMI, hypertension, diabetes, education level, marital status, ratio of family income to poverty, smoking, and drinking. *P < 0.05, **P < 0.01, ***P < 0.001. [file Image11.tif]
